# The Removal of Naso-Pharyngeal Fibromata by the Galvano-Cautery, or Steel Wire Écraseur

**Published:** 1884-06

**Authors:** E. Fletcher Ingals

**Affiliations:** Chicago


ARTICLE IV.

### THE REMOVAL OF NASO-PHARYNGEAL FIBROMATA BY THE GALVANO-CAUTERY, OR STEEL WIRE ÉCRASEUR. By E. FLETCHER INGALS, A.M.M.D., Chicago.

Naso-pharyngeal fibromata usually have their origin in the periosteum which covers that portion of the base of the skull forming the roof of the naso-pharnyx.

These tumors may extend downward into the mouth, forward into the nasal cavity, or upward, perforating the cranium and pressing upon the brain.

When large, they not infrequently send off prolongations into the maxillary sinus, and in some cases the frontal and sphenoidal sinuses are involved, consequently in some instamces great deformity of the face results.

Owing to the obstruction thus caused, respiration and deglutition may be seriously interfered with, and in consequence of the pressure, the senses of hearing, sight, and smell are more or less impaired.

The growths are hard to the touch, and are usually rounded, having a smooth surface, slightly lighter in color than the surrounding mucous membrane; sometimes, however, and particularly when the nasal cavities are involved, these tumors are lobulated in form.

Some of them grow slowly, but in other cases they increase in size with great rapidity; and, unfortunately they are quite liable to return after removal.

Aside from the symptoms already referred to in connection with the special senses, they may cause severe pain, frequent epistaxis, and constant catarrhal discharge. They eventually disturb the sleep and prevent the patient from taking sufficient food; exhaustion supervenes, and ultimately if not removed, they are liable to prove fatal.

Various forms of treatment, such as electrolysis, the local application of chemical agents, have been tried, but nothing short of a complete destruction of the growth by operative measures can give satisfactory results.

The operation usually recommended by surgeons, consists in tearing or gouging out the tumor after access to it has been obtained, by removal of the hard and soft palate, or of the superior maxillary bone, the latter being the procedure generally resorted to; but within a few years rhinogologists have demonstrated the practicability of a much safer operation, which has thus far been attended with better results than when the maxilla, or palate, has been removed.

This operation is performed with the galvano-cautery, or steel wire écraseur, the loop being passed through the nostrils in some cases, and in others through the mouth and up behind the soft palate.

Within the past two years I have operated on five cases of naso-pharyngeal tumors by this latter method, and with better results than could have been obtained by excision of a part or the whole of the superior maxillary bone.

In four of these cases over a year and a half have now elapsed since the last operation, as their histories will show. In the fifth the growth appeared microscopically of a semi-malignant type. It is not here reported, but the tumor has been removed, and is now growing again; however, owing to the patient's great improvement in health, and to the histories of two of the other four cases, which I have heard from lately, I do not despair of ultimately curing the patient.

CASE I.—Fibroma. In May, 1881, I operated upon the first case of this kind which had come under my observation.

The patient, J. W., æt. 21, stated that about twenty months previously he had contracted a severe cold, since when he had been unable to breathe through the right nostril, excepting with forced respiration, and then but slightly. The left nostril had also been occluded whenever he had a cold, but at other times it had been comparatively free.

Rhinoscopic examination revealed a large ovoid tumor blocking the right posterior naris and overlying about half of the left. With the galvano-cautery écraseur I removed the larger part of the growth and would have secured all, had not the current failed me so that I was obliged to readjust the wire. The bleeding prevented me from doing this perfectly.

Prof. I. N. Danforth, of Chicago, made a microscopic examination of the tumor, and pronounced it a small celled-sarcoma, the most benignant of the fibromata.

A few weeks later the remainder of the growth was perfectly removed by the galvano-cautery écraseur.

Sixteen months later I found a small tumor at the seat of the old growth, about the size of a hazel-nut. This I removed with Jarvis' écraseur.

At the present time, three years after the original operation, a small sessile swelling may be seen at the posterior end of the upper turbinated bone. This presents no point where the écraseur could be made to hold, and as it has enlarged very slowly, I have not attempted to cauterize it.

CASE II.—Fibroma. H. L., aged 19. Was brought me by my friend, Dr. F. E. Olney, of Warsaw, Ind. I found a large naso-pharyngeal growth, which presented slightly below the soft palate, and filled the naso-pharyngeal space.

It had been annoying him very much for twelve months; preventing nasal respiration and causing profuse discharges from the nose and pharynx. The tumor was so large that I found it impossible to pass Bellocq's canula or an Eustachian catheter through the nares; however, after much difficulty, I succeeded in passing a small, soft rubber catheter. In doing this, all excepting half an inch of the distal end of the catheter had been kept rigid by the stilet so that when pressure was made the flexible end insinuated itself between the growth and the palate and was finally forced into the pharynx and brought out through the mouth. A string was then attached to it and drawn back through the nose, by which means an elastic band was drawn through the naris to tie the soft palate forward; and subsequently a loop of platinum wire was drawn back through the naris into the mouth.

The loop was opened, and by patient manipulation for about three hours, it was carried up until it encircled the base of the tumor, close to the bone from which it sprang. The ends of the platinum wire were then passed through the tubes of an electrode which was forced back along them through the nose until the tumor was tightly ensnared.

The handle with which the conductors from the battery were connected, was then attached, the platinum wires fixed to the ratchet and the current turned on; but I soon found that a short circuit was established through the metallic axle of the ratchet wheel, so that the pedicle could not be burned. I then removed the ratchet and attached the wire to a lead pencil, by means of which traction was made. The current then passed through the loop, heated the wire, and the pedicle was speedily cut and the growth ejected from the mouth.

Because of failure of the ratchet the base of the tumor was accidentally divided much quicker than I wished, and as a consequence profuse bleeding ensued; this, however, was checked in a short time by the insufflation of tannin behind the palate.

The tumor was slightly nodular in form, and measured one and one-half inches in its antero-posterior diameter, by one and three-fourths inches in its vertical diameter.

It had been cut off close to the basilar process of the occipital bone to which it had been attached by a pedicle three-quarters of an inch in diameter.

The patient returned to his home the same evening. The cut surface healed over promptly, and for two months he seemed perfectly well, but at the end of that time Dr. Olney noticed that the tumor was re-appearing. It grew rapidly for about two months, when it had attained about one-third its former size, since when it has remained stationary.

The patient's general health has been perfect for the last eighteen months, and there has been no increase in the size of the tumor, but it will be again removed as soon as the patient can spare the time, and then the stump will be thoroughly cauterized.

CASE III.—Fibroma. A charity patient. J. H., æt. 26, came to me during the latter part of the winter of 1882-83, complaining only of a slight cold. I noticed a peculiar lack of nasal resonance in his voice which directed my examination to the naso-pharynx, where I found a tumor about the size of a hickory-nut, which from its position and from the movements that could be given to it with a probe seemed to be attached to the upper portion of the posterior border of the vomer.

With a modification of Jarvis' ècraseur, half an inch of the end of which had been bent up at nearly a right angle, I passed a steel wire behind the palate and ensnared the tumor and cut it off.

The tumor presently dropped into the mouth and was ejected. It had the form of a flattened sphere, measuring one inch in its long diameter by one-half inch in thickness. Dr. E. P. Davis, of this city, examined the growth microscopically, and reported it almost wholly fibrous. Afterward a similar but smaller growth was detected in the posterior part of the left nasal cavity, which subsequently came down into the naso-pharynx. I made one attempt to catch it in the snare, but failing on the first trial, I was obliged to defer the operation for want of time, and the patient did not return.

CASE IV.—Fibro-Myxoma. A. P., æt. 32, machinist, came to my clinic at the Central Dispensary in December, 1882. He gave the following history: When two years of age his mother had noticed something growing in his nose, but nothing was done for it until he was fourteen years of age, when it was operated on by forceps, and a large mass removed.

Subsequently he was free from the trouble for several years, but at the age of nineteen it had returend soas to necessitate another operation.

From that time until about a year before coming to me he had experienced no difficulty in breathing through the nose.

Upon examination, I found a large growth completely filling the right nasal cavity, and closing the left by pressure; and causing considerable deformity of the nose.

It also passed back and filled about half the naso-pharynx.

After several futile attempts to tear away the anterior portion of this tumor with forceps, about a third of it was cut off with the galvano-cautery wire loop. Finally, with a modification of Jarvis' snare, all the growth was removed. In this case it was impossible to engage any considerable portion of the tumor at once, as the instrument could not be passed through the nose until most of the growth had been taken out; therefore, at each sitting, I removed two or three pieces about the size of filberts, and then allowed the patient to go. Dr. E. A. Davis found this to be a fibro-myxoma.

At the meeting of the American Laryngological Association held in New York, in May, 1883, Dr. Rufus P. Lincoln presented three cases which he had operated upon successfully by the galvano-cautery écraseur. For the sake of comparison, he also gave a tabulated statement of the results of seventy-four operations for naso-pharyngeal tumors which he had found reported.

The statement which has been furnished me by Dr. Lincoln is substantially as follows:

The seventy-four operations were performed on fifty-eight patients.

Thirty-nine operations were done upon twenty-eight patients by section of the facial bones or lying open of cicatrices resulting from previous operations of the same character. Of these, in thirteen, the record ceased shortly after the operation, and the final results were unknown. Of the fifteen remaining, eight died from the operation, and in three others it nearly proved fatal, while in only four was there no return of the tumor within a year.

Seven operations were done on seven patients by scissors, knife or forceps. One of these died, five were lost sight of, or no subsequent report was made, and in only one are we told there was no recurrence within a year.

Two cases had been treated with injections of chloride of zinc, or cauterization by the same agent. In one the tumor returned, the other case was lost sight of. Three cases were treated by electrolysis. One did not recur within a year; the others disappeared. Of these forty cases, in twenty-one the history after the operation was incomplete, and probably not favorable to the operation.

Nine, nearly one-fourth of all the cases, or about one-half of those in whom the history was complete, died, and only seven remained well for one year.

Contrasting with these the cases operated upon by the ordinary écraseur, or ligation, we find that of eleven operated on none were fatal, two were lost sight of, and of those remaining there was no recurrence at the end of a year in four cases, which is a very much better showing than for the preceding operations.

Nine have been operated on by the galvano-cautery écraseur, and one by the actual cautery. None of them were fatal; two were lost sight of, and six were well at the end of a year, results infinitely superior to those of any of the other methods.

The remarkable success of this form of treatment is supposed to have been due to thorough cauterization of the stumps of the tumors, which was practiced in several instances.

In my own cases, the stumps of the tumors have not been cauterized, but although there has been some new growth in the two still under observation, there has been no enlargement for over a year in one, and over two years in the other; and neither now gives the patient any discomfort.

In two others, occurring in dispensary patients, the disagreeable symptoms having been removed, it is probable that I will not see them again until the tumors have re-grown sufficiently to be annoying. One of these was cured at the time. In the other the operation was incomplete.

From my experience in these cases, I believe that either with the galvano-cautery or with the steel wire écraseur the great majority of these tumors may be completely removed, and, as has been shown by Dr. Lincoln, if their bases are thoroughly cauterized with a hot wire, they are not likely to recur. As illustrated by two of my cases, although they often recur the subsequent growth may be very small in comparison with the first, and after a short time it may become quiescent.

The results thus far are certainly in favor of this method of operating in most cases of naso-pharyngeal fibromata over that usually advised by surgeons. The advantages are, that this leaves no scar: the tumors are less likely to recur, and the risks to the patient are infinitely less.

